# Ras-Related C3 Botulinum Toxin Substrate 1 Combining With the Mixed Lineage Kinase 3- Mitogen-Activated Protein Kinase 7- c-Jun N-Terminal Kinase Signaling Module Accelerates Diabetic Nephropathy

**DOI:** 10.3389/fphys.2021.679166

**Published:** 2021-06-14

**Authors:** Changjiang Ying, Jiao Dai, Gaoxia Fan, Zhongyuan Zhou, Tian Gan, Yusheng Zhang, Yuanjian Song, Xiaoyan Zhou

**Affiliations:** ^1^Department of Endocrinology, Affiliated Hospital of Xuzhou Medical University, Xuzhou, China; ^2^The Graduate School, Xuzhou Medical University, Xuzhou, China; ^3^Department of Genetics, School of Life Sciences, Xuzhou Medical University, Xuzhou, China

**Keywords:** Ras-related C3 botulinum toxin substrate 1, MLK3-MKK7-JNK signal module, diabetic nephropathy, c-Jun N-terminal kinase, apoptosis

## Abstract

Ras-related C3 botulinum toxin substrate 1 (RAC1) activation plays a vital role in diabetic nephropathy (DN), but the exact mechanism remains unclear. In this study, we attempted to elucidate the precise mechanism of how RAC1 aggravates DN through cellular and animal experiments. In this study, DN was induced in mice by intraperitoneal injection of streptozotocin (STZ, 150mg/kg), and the RAC1 inhibitor NSC23766 was administered by tail vein injection. Biochemical indicators, cell proliferation and apoptosis, and morphological changes in the kidney were detected. The expression of phosphorylated c-Jun N-terminal kinase (p-JNK), nuclear factor-κB (NF-κB), and cleaved caspase-3 and the interaction between RAC1 and the mixed lineage kinase 3 (MLK3)-mitogen-activated protein kinase 7 (MKK7)-JNK signaling module were determined. Furthermore, the colocalization and direct co-interaction of RAC1 and MLK3 were confirmed. Our results showed that RAC1 accelerates renal damage and increases the expression of p-JNK, NF-κB, and cleaved caspase-3. However, inhibition of RAC1 ameliorated DN by downregulating p-JNK, NF-κB, and cleaved caspase-3. Also, RAC1 promoted the assembly of MLK3-MKK7-JNK, and NSC23766 blocked the interaction between RAC1 and MLK3-MKK7-JNK and inhibited the assembly of the MLK3-MKK7-JNK signaling module. Furthermore, RAC1 was combined with MLK3 directly, but the RAC1 Y40C mutant inhibited the interaction between RAC1 and MLK3. We demonstrated that RAC1 combining with MLK3 activates the MLK3-MKK7-JNK signaling module, accelerating DN occurrence and development, and RAC1 Y40 is an important site for binding of RAC1 to MLK3. This study illustrates the cellular and molecular mechanisms of how RAC1 accelerates DN and provides evidence of DN-targeted therapy.

## Introduction

Diabetic nephropathy (DN), the most common microvascular complication of diabetes, occurs in approximately 40% of diabetics and has become a leading cause of end-stage renal disease globally (Zhang et al., 2019; Chang et al., 2020; Martinez-Moreno et al., 2020). Published studies suggest that activation of Ras-related C3 botulinum toxin substrate 1 (RAC1) induces renal damage and plays an important role in the pathogenesis and progression of DN (Lin et al., 2015; Maier et al., 2018).

Ras-related C3 botulinum toxin substrate 1 is a member of the Rho family of small GTPases and plays an important role in inflammation and apoptosis (Sahajpal et al., 2019; Hirohama et al., 2020). RAC1 signaling promotes an immune response and apoptosis (Kowluru, 2020). Previous research has shown that RAC1 is significantly enhanced in kidneys induced by hyperglycemia stimulation, which activates its downstream signaling pathways leading to glomerular cell damage and apoptosis (Lv et al., 2018). Inhibition of RAC1 activation significantly reduces podocyte apoptosis and alleviates renal pathological changes (Lin et al., 2008). Previous studies have demonstrated that the specific deletion of RAC1 could reduce the damage of diabetic renal tissue and delay DN progression (Veluthakal et al., 2016; Kim et al., 2019). Nevertheless, the precise mechanism underlying RAC1 promotion of DN remains unclear.

Evidence supports the idea that RAC1 as an upstream signaling molecule could activate the c-Jun N-terminal kinase (JNK) pathway resulting in cellular apoptosis (Brown et al., 2006; Tebar et al., 2020), but how RAC1 regulates JNK activation needs further exploration. JNK belongs to the mitogen-activated protein kinase (MAPK) family and is typically involved in DN as a critical mediator (Wang et al., 2015; Zhou et al., 2018). JNK phosphorylation is controlled by the three-enzyme cascade reaction mixed lineage kinase 3 (MLK3)-MAPK mitogen-activated protein kinase 7 (MKK7)-JNK (He et al., 2016; Lin et al., 2017). Moreover, the support protein, JNK-interacting protein 1 (JIP1), is also required to provide anchor sites to combine MLK3-MKK7-JNK and then enhance JNK phosphorylation and regulate JNK activity (Bosco et al., 2009). A previous study suggested that knockout of RAC1 can reduce JNK phosphorylation either in cells under high glucose conditions or streptozotocin (STZ)-induced diabetic models (DMs; Lv et al., 2018). However, whether RAC1 promotion of JNK phosphorylation to induce DN is associated with the MLK3-MKK7-JNK signaling module and the specific molecular mechanism remains unclear.

## Materials and Methods

### Reagent

Streptozotocin was obtained from Sigma, and the RAC inhibitor, NSC23766, was supplied by Selleck. DMEM medium (Cat. No. KG075844) and trypsin containing 0.25% EDTA were purchased from Keygen Biotech, and fetal bovine serum (FBS) was obtained from Hangzhou Sijiqing Biological Engineering Materials Co., Ltd. Cell proliferation assay kit, cell counting kit-8 (CCK-8; Cat. No. C0038), was provided by Beyotime Biotechnology and Annexin V-7 AAD/PI apoptosis assay (Cat. No. 559763) was purchased from BD Biosciences. RAC1 (Cat. No. 66122-1-lg), MLK3 (Cat. No. 11996-1-AP), MKK7 (Cat. No. 55030-1-AP), JNK (Cat. No. 51151-1-AP), JIP1 (Cat. No. 14568-1-AP), H3 (Cat. No. 17168-1-AP), IgG (Cat. No. B900620), Alexa Fluor 488-conjugated Affinipure Goat Anti-Rabbit IgG (Cat. No. SA00013-2), Alexa Fluor 594-conjugated Goat Anti-Mouse IgG (Cat. No. SA00013-3), β-actin (Cat. No. 60008-1-Ig; Cat. No. 51151-1-AP), GST Tag Rabbit polyclonal antibody (Cat. No. 10000-0-AP), and His-Tag Mouse monoclonal antibody (Cat. No. 66005-1-lg) were purchased from Proteintech. p-JNK antibody (Cat. No. 9255), NF-κB antibody (Cat. No. 8242), and cleaved caspase-3 (Cat. No. 9255) were obtained from Cell Signaling Technology. Bimethylformate (BCA) protein concentration detection kits (Cat. No. P0010), PMSF, RIPA (Cat. No. KGP703-100), and DAPI were purchased from Beyotime Biotechnology. Urine protein quantitative test box (Cat. No. C035-2), blood urea nitrogen (BUN) test box (Cat. No. C013-2-1), and creatinine (CR) test kit (Cat. No. C011-2-1) were obtained from Nanjing Jiancheng Bioengineering Research Institute (Nanjing, China). Pierce GST Protein Interaction Pull-Down Kit (Cat. No. 21516) was purchased from Thermo Fisher Scientific, and the TransIn EL Transfection Reagent (Cat. No. DP108) was purchased from TransGen Biotech Company (Beijing, China).

### Cell Experimental Design

HEK293T cells were seeded at 1 × 10^5^ cells/ml in 96-well plates at 100 μl/well. After growing to log phase, the cells were cultured in serum-free DMEM low glucose medium for 24 h (synchronized) before randomly dividing into the normal group (NG; 25 mM glucose), the high glucose group (HG; 80 mM glucose), the high glucose + RAC1 inhibitor group (HG + NSC; 80 mM glucose + 10 μM NSC23766), the high glucose + solvent control group (HG + DMSO), or the mannitol group (MG; 25 mM glucose + 55 mM mannitol as an osmotic pressure control).

### Cell Proliferation Assay

HEK293T cells were seeded in 96-well plates with DMEM containing 25 mM glucose and 10% FBS. Once the cell confluency reached 80%, the medium was replaced with DMEM containing only 25 mM glucose. CCK-8 solution was added after 24 h, and the cells were incubated for a further 2–4 h. Absorbance was determined with a microplate reader (Thermo, Massachusetts, United States) at 450 nm.

### Cell Transfection

HEK-293 T cells were transfected at 80% confluency with appropriate plasmids using EL transfection reagent according to the instructions of the manufacturer. After incubation with plasmids, cells were treated with 25 mM glucose for an additional 48 h.

### Flow Cytometric Analysis

HEK293T cells were seeded at 5 × 10^5^ cells/ml in 6-well plates at 2 ml/well. After intervention for 48 h according to the experimental design, all groups were digested with trypsin (without EDTA), centrifuged, resuspended, and then 1 × 10^6^/100 μl of cells were removed to a flow tube. After washing the cells three times, 5 μl of Annexin V and 5 μl of 7 AAD were added, the cells were gently vortexed, incubated at room temperature for 15 min in the dark, and analyzed by flow cytometry to detect the apoptosis ratio of cells.

### Experimental Animals

Eight-week-old male Kunming mice were purchased from the Animal Experimental Center of Xuzhou Medical University (Permit Number: SYXK (SU) 2010-0011). Experimental mice were kept in a bacteria-free environment at a room temperature of 25 ± 1°C and 55–65% humidity, with free access to food and water. After 1 week of adaptive feeding, mice were randomly divided into normal (*n* = 30) and diabetic model (DM; *n* = 54) groups. After fasting for 16 h, the DM group was injected intraperitoneally with STZ at a dose of 150 mg/kg (Breyer et al., 2005). Mice in the normal group were injected intraperitoneally with an equal volume of sodium citrate buffer. After 72 h, tail vein blood glucose was measured with a blood glucose meter. The random blood glucose was ≥16.7 mmol/L as diabetic mice and was employed in subsequent experiments.

Nine-week-old diabetic mice were divided randomly into the DM group, DM + NSC group (intraperitoneal injection of NSC23766 at 1.5 mg/kg/day for 12 weeks), and DM + DMSO solvent control group (intraperitoneal injection of DMSO at 1.5 mg/kg/day for 12 weeks). Normal mice were assigned to the control group (Con) and normal mice treated with NSC23766 inhibitor group (Con + NSC; intraperitoneal injection of NSC23766 at 1.5 mg/kg/daily). At 21-week-old, mice were anesthetized with 1% pentobarbital (50 mg/kg) for subsequent studies.

### Assessment of Blood Lipids and Renal Function

Mouse urine collection over 24 h was performed using metabolic cages to assay 24 h urine albumin (24 h-UP). Urinary albumin was determined using a commercial kit. Blood glucose levels were tested using tail vein blood. Serum was obtained following centrifugation of heart blood. Triglyceride (TG), low-density lipoprotein (LDL), high-density lipoprotein (HDL), BUN, serum creatinine (Scr), and total cholesterol (TC) were detected using a specific kit. All operations were strictly performed according to the instructions of the manufacturers.

### Hematoxylin-Eosin Staining

Mouse kidneys were removed and fixed with 4% paraformaldehyde for 48 h at 4°C. After embedding in paraffin, the samples were sliced into 6 μm-thick sections, deparaffinized with fresh xylene, and rehydrated with a gradient of ethanol. After staining with H&E, pathological and morphological observations were carried out with a microscope.

### Transmission Electron Microscopy and Morphometry

Small pieces of renal cortex were immersed in 2.5% glutaraldehyde overnight and postfixed with 1% osmium tetroxide at 4°C for 2 h. After thoroughly rinsing the tissues using distilled water, the specimens were dehydrated with an ethanol gradient. Finally, the samples were embedded in molds with epoxy resin. Ultrathin sections were stained with uranyl acetate and lead citrate and then viewed using a transmission electron microscope. The thickness and foot depth of the substrate were measured and calculated using the ImageJ image analysis system. A plurality of points was randomly selected from each basement membrane, and the thickness of the basement membrane at each point and the width of the foot process were measured in cm for statistical analysis.

### Immunofluorescence

Fixed cells were permeabilized in 0.3% Triton X-100 for 30 min and then blocked with 10% goat serum for 1 h. After incubation with rabbit anti-MLK3 antibody and mouse anti-RAC1 at 4°C overnight, the fixed cells were incubated with Alexa Fluor 594-conjugated donkey anti-rabbit antibody and Alexa Fluor 488-conjugated donkey anti-mouse (Proteintech) for 2 h. After staining with 0.1% DAPI (Beyotime), the cells were observed and analyzed under a confocal laser scanning microscope.

### Co-immunoprecipitation

Protein samples were removed from the 80°C refrigerator and put on ice. Once each sample was completely thawed, 800 mg of protein was prepared per sample. Each sample was incubated for 1–2 h with 4 μg of Protein A primary antibody at 4°C, then 40 μl of resuspended Protein A/G agarose was added. Tubes were capped and incubated at 4°C on a rotating device overnight. Immunoprecipitates were collected by centrifugation at 3,000 *g* for 5 min at 4°C. The pellets were washed three times with 500 ml of IP buffer, centrifuging at 3,000 *g* for 5 min at 4°C. After the final wash, the supernatants were discarded, and pellets were resuspended in 20–25 μl of 2x electrophoresis sample buffer. Samples were boiled for 10 min before cooling to room temperature and centrifuging at 3,000 *g* for 5 min. Supernatants were absorbed from the samples and stored in a new EP pipe. Electrophoresis and immunoblotting were performed as described under the western blotting subsection.

### GST Pull-Down

*Escherichia coli* was transformed with appropriate plasmids and cultured overnight. GST fusion protein expression was induced with isopropyl β-D-thiogalactoside (IPTG). GST pull-down experiments were performed according to the instructions of the manufacturer. GST fusion proteins were mixed with equilibrated glutathione agarose resin and incubated for 2 h at 4°C. After five washes in TBS containing pull-down lysis buffer, the reaction products were incubated with bait protein for 2 h and washed five times with TBS containing pull-down lysis buffer. Finally, the proteins bound to the GST-RAC1 particles were washed with elution buffer and solubilized in a 5x loading buffer. Protein samples were analyzed by sodium dodecyl-sulfate polyacrylamide gel electrophoresis (SDS-PAGE).

### Western Blotting

Cells or renal cortex were homogenized in ice-cold RIPA lysis buffer with added protease inhibitors before centrifugation at 14,000 rpm for 30 min at 4°C. Protein concentration was determined using a BCA protein concentration assay kit. Equal amounts of protein samples were electrophoresed at 80 V through 10 or 12% SDS-polyacrylamide gels depending on the size of the target proteins and then transferred to nitrocellulose membrane. After blocking for 2 h with 5% nonfat milk, the nitrocellulose membranes were incubated with the appropriate primary antibodies (anti-JNK, anti-p-JNK, anti-NF-κB, anti-cleaved caspase-3, and anti-β-actin) at 4°C overnight. Then, the blots were incubated with anti-mouse or anti-rabbit IgG conjugated to horseradish peroxidase for 2 h at room temperature. Immunoreactive bands were scanned using the Odyssey Infrared Imaging System.

### Statistical Analysis

Values were reported as mean ± SD. Data were analyzed using SPSS 17.0 software. Statistical significance between the two groups was analyzed by the Student’s *t*-test. A one-way ANOVA was used to compare multiple groups. Value of *p* < 0.05 was considered statistically significant.

## Results

### Cell Proliferation and Apoptosis

**Figure 1:**
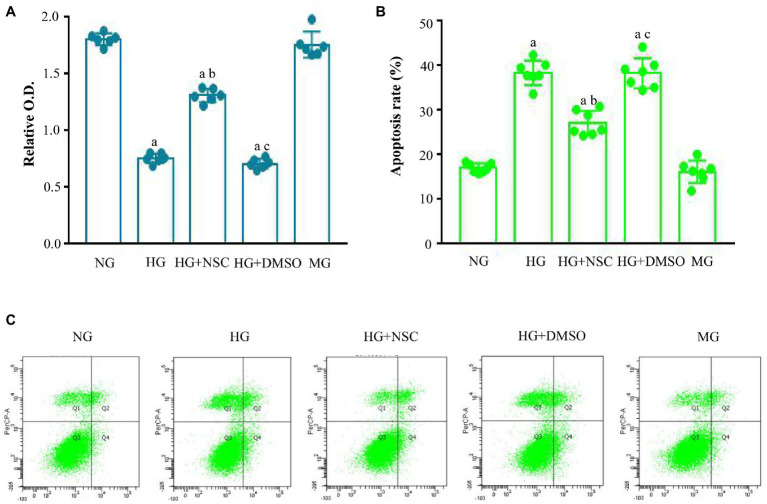
Effect of Ras-related C3 botulinum toxin substrate 1 (RAC1) on cell proliferation and apoptosis. **(A)** Cells incubated in different conditions were analyzed using cell counting kit-8 (CCK-8). **(B)** Typical flow cytometry plots. **(C)** Analysis of cellular apoptosis rate. Data are shown as mean ± SD. ^a^*p* < 0.05 vs. normal group (NG); ^b^*p* < 0.05 vs. high glucose group (HG); ^c^*p* < 0.05 vs. high glucose + RAC1 inhibitor group (HG + NSC). *n* = 7 in each group.

Cell proliferation was lower in the HG group than in the Con group; however, treatment with NSC23766 significantly increased cell proliferation in the HG + NSC group (Figure 1A). These results show that the RAC1 inhibitor NSC23766 enhanced cell proliferation in a high glucose environment. Next, we detected cell apoptosis using flow cytometry. Compared with the HG group, cell apoptosis was significantly decreased in the NG and HG + NSC groups, indicating that inhibition of RAC1 reduces cell apoptosis induced by high glucose levels (Figures 1B,C).

### Biochemical Indexes

**Figure 2:**
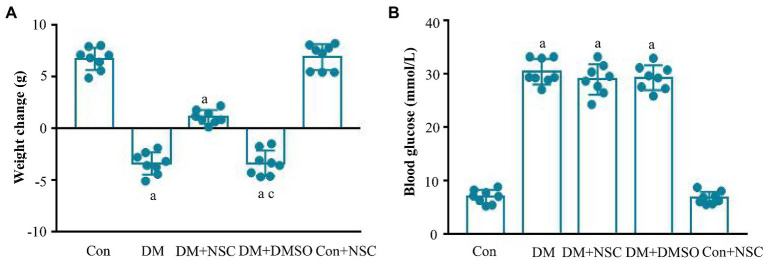
Biochemical indexes. **(A)** Evaluation of body weight in each group. **(B)** Detection of blood glucose level in each group. Data are shown as mean ± SD. ^a^*p* < 0.05 vs. Con group; ^b^*p* < 0.05 vs. DM group; ^c^*p* < 0.05 vs. DM + NSC group; Con, normal control group; DM, diabetes group; DM + NSC, inhibitor group; DM + DMSO, solvent control group; Con + NSC: drug control group. *n* = 8 in each group.

To evaluate the role of RAC1 in DN, we administered RAC1 inhibitor NSC23766 to diabetic mice. Mouse body weight loss was significantly decreased in the DM + NSC group compared with the DM group (Figure 2A). No remarkable differences in blood glucose were observed between the DM, DM + NSC, and DM + DMSO groups (Figure 2B). TG, LDL, BUN, Scr, TC, and 24 h-UP were higher in diabetic mice than in normal mice, but TG, LDL, BUN, and Scr were lower in the DM + NSC group than in the DM group. HDL was decreased in the DM group compared with the Con group; however, RAC1 inhibitor NSC23766 increased HDL in the DM + NSC group (Table 1). These data suggest that RAC1 inhibitor, NSC23766, improves weight loss, blood lipid levels, and renal function in diabetic mice.

**Table 1:** Biochemical and renal function parameters.

Groups	TG (mmol/L)	LDL (mmol/L)	HDL (mmol/L)	BUN (mmol/L)	Scr (μmol/L)	TC (mmol/L)	24 h-UP (mg)
Con	0.81 ± 0.18	0.32 ± 0.03	1.87 ± 0.04	7.6 ± 1.99	8.7 ± 1.32	4.13 ± 1.09	17.13 ± 2.79
DM	2.53 ± 0.24^a^	0.67 ± 0.05^a^	0.95 ± 0.07^a^	16.7 ± 3.43^a^	22.6 ± 4.17^a^	9.28 ± 2.13^a^	92.57 ± 7.43^a^
DM + NSC	1.77 ± 0.23^a^^,^^b^	0.54 ± 0.05^a^^,^^b^	1.67 ± 0.05^a^^,^^b^	11.2 ± 2.17^a^^,^^b^	14.8 ± 2.32^a^^,^^b^	6.23 ± 1.79^a^	83.68 ± 6.79^a^
DM + DMSO	2.41 ± 0.27^a^^,^^c^	0.66 ± 0.05^a^^,^^c^	1.03 ± 0.08^a^^,^^c^	16.3 ± 3.72^a^	23.1 ± 4.73^a^^,^^c^	8.87 ± 1.96^a^	95.87 ± 8.96^a^
Con + NSC	0.91 ± 0.18	0.35 ± 0.04	1.85 ± 0.05	8.40 ± 2.14	9.40 ± 2.13	3.56 ± 1.12	18.56 ± 6.12

Values represent means ± SD. *n* = 8 in each group.

a *p* < 0.05 vs. normal control group (Con).

b *p* < 0.05 vs. diabetes modal group (DM).

c *p* < 0.05 vs. inhibitor group (DM + NSC).

### Renal Morphology and Ultrastructure

**Figure 3:**
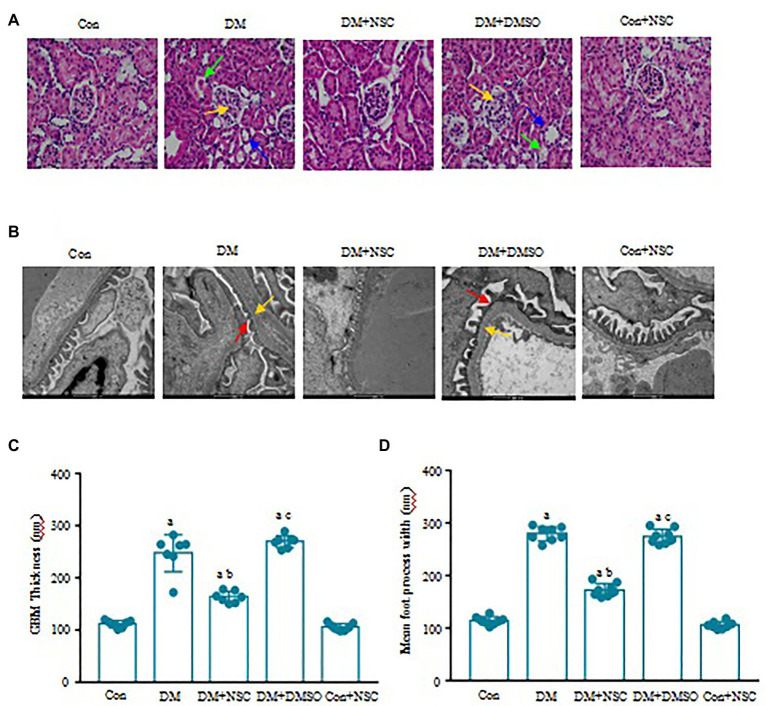
Effect of RAC1 inhibition on glomeruli. **(A)** Assessment of renal morphological changes. Representative photomicrographs of H&E staining are shown. Yellow arrows show glomerular capillary disorder, blue arrows show renal tubule atrophy, and green arrows show vacuolar degeneration. The scale bar is 50 μm (magnification × 40). **(B)** Representative photographs of glomeruli. Red arrows show chorion fall out, and yellow arrows show basement membrane. The scale bar is 500 nm (magnification × 4,000). **(C)** The thickness of the glomerulus basement membrane in each group. **(D)** Width of the foot processes. Values denote the mean ± SD. ^a^*p* < 0.05 vs. the Con group; ^b^*p* < 0.05 vs. the DM group; ^c^*p* < 0.05 vs. the DM + NSC group. *n* = 7 from four mice in each group.

Evidence shows that renal glomerular basement membrane (GMB) extension and thickening and tubule atrophy are marked pathological changes in the diabetic model (Ying et al., 2017). In the DM group, morphometric analysis of HE-stained kidney sections revealed significant renal tubule atrophy, vacuolar degeneration, and glomerular capillary disorder (Figure 3A). Inhibiting the activity of RAC1 could improve pathological changes in DN. Hyperglycemia exacerbated chorion falling out and basement membrane turbulence in tubules, aggravated the foot process fusion, and affected GMB thickness and extension in the glomerulus (Figures 3B–D). Also, inhibition of RAC1 reduced the renal injury. These results indicate that inhibition of RAC1 alleviates morphological and ultrastructure disorders of the kidney in diabetic mice.

### Inhibition of RAC1 Reduces the Expression of P-JNK, NF-κB, and Cleaved Caspase-3

**Figure 4:**
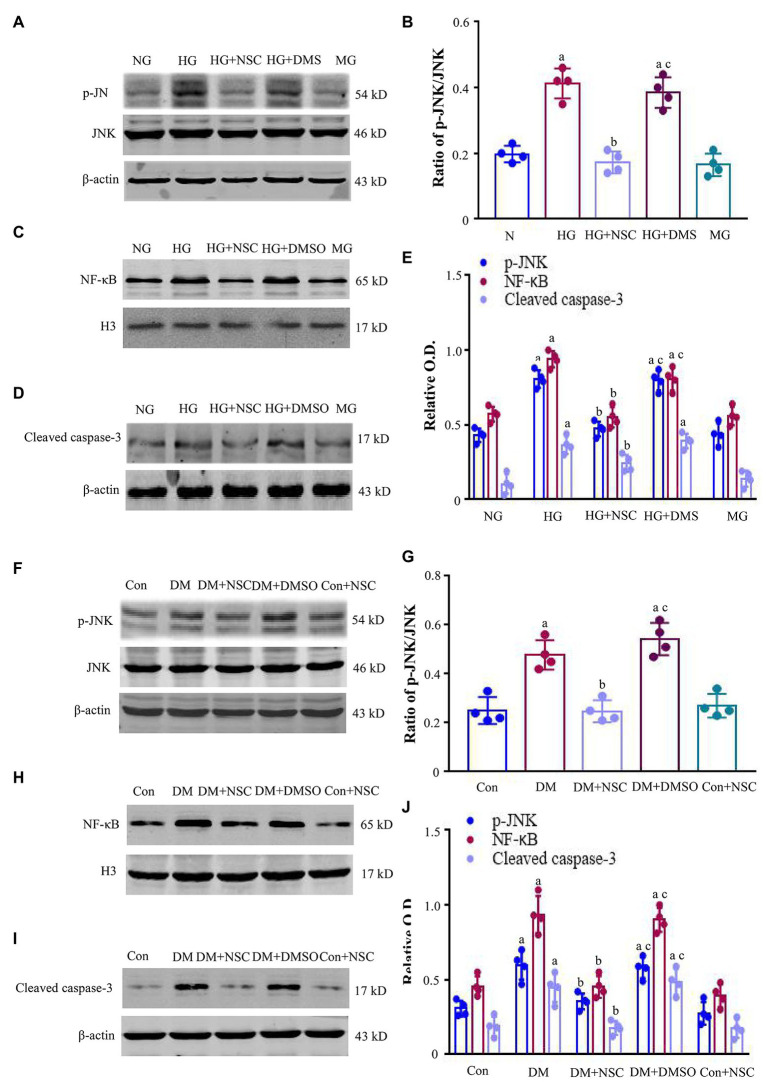
Expression of c-Jun N-terminal kinase (JNK), nuclear factor-κB (NF-κB), and cleaved caspase-3. **(A)** Typical bands of phosphorylated c-Jun N-terminal kinase (p-JNK) and JNK were shown. **(B)** The ratio of p-JNK/JNK was analyzed. **(C,D)** Typical bands of NF-κB and cleaved caspase-3 in HEK-293 T cells. **(E)** Analysis of optical density (OD) values of protein expression. **(F)** Representative bands present the expression of p-JNK and JNK. **(G)** The ratio of p-JNK/JNK was analyzed. **(H,I)** Representative bands showing the levels of NF-κB, and cleaved caspase-3 in renal tissues. **(J)** Bands calculated by OD analysis. Results are presented as mean ± SD. ^a^*p* < 0.05 vs. the NG group **(B,E)** and the Con group **(G,J)**; ^b^*p* < 0.05 vs. the HG group **(B,E)** and the Con group **(G,J)**; ^c^*p* < 0.05 vs. the HG + NSC group **(B,E)** and the DM + NSC group (**G** and **J**). *n* = 4 in each group.

To clarify the possible mechanisms of RAC1 in DN, we detected the levels of p-JNK, NF-κB, and cleaved caspase-3. The expression of p-JNK was increased in the HG group compared with the NG group (Figures 4A,B). The HG + NSC group had lower total p-JNK levels than the DM group. The ratio of p-JNK/JNK in the HG group is higher than the NG group, but NSC23766 reduced this ratio (Figures 4A,B). Likewise, the trends of NF-κB and cleaved caspase-3 were consistent with that of p-JNK (Figures 4C–E). The expression of p-JNK, NF-κB, and cleaved caspase-3 proteins were remarkably higher in diabetic mice than in normal mice, and NSC23766 inhibited p-JNK signal pathway activation (Figures 4F–J). Together, these findings support the concept that RAC1 aggravates DN by activating the JNK-related signaling pathway to promote apoptosis.

### Co-precipitation of RAC1 and MLK3-MKK7-JNK Signaling Module

**Figure 5:**
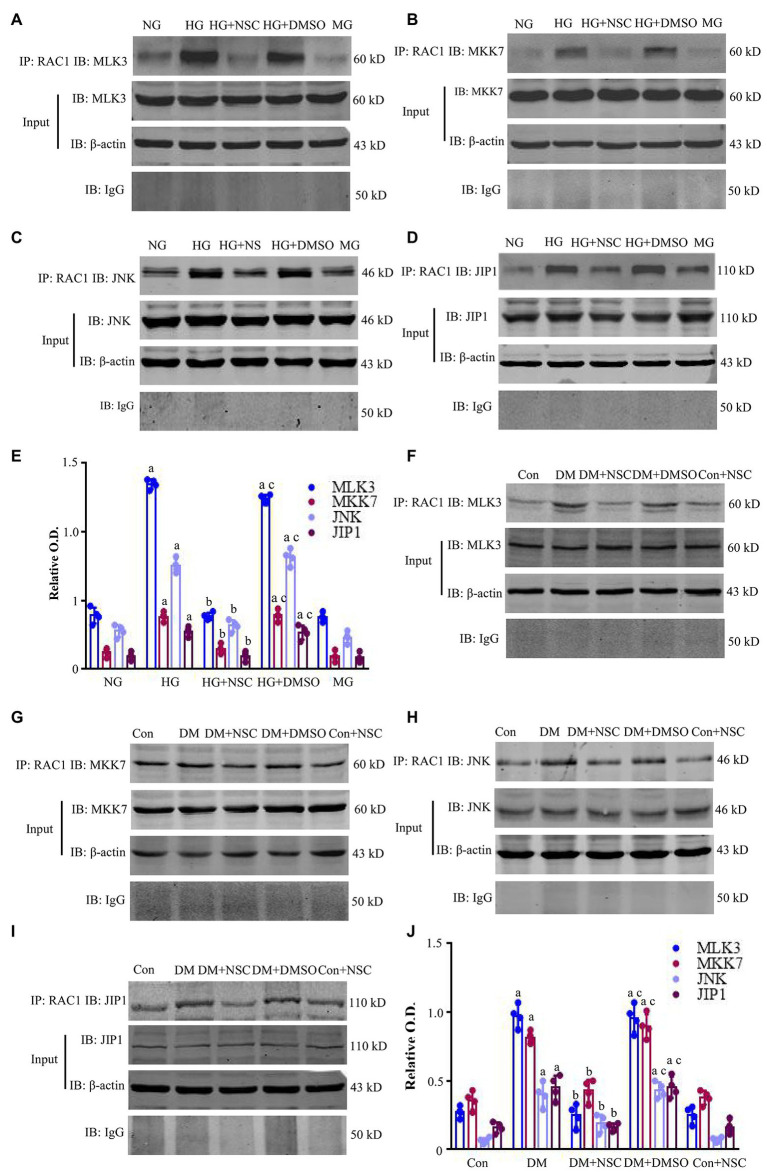
Co-immunoprecipitation (Co-IP) of RAC1 and the mixed lineage kinase 3- mitogen-activated protein kinase 7- c-Jun N-terminal kinase (MLK3-MKK7-JNK) signaling module. **(A–D)** Co-IP showing the interactions between RAC1 and MLK3, MKK7, JNK, and JNK-interacting protein 1 (JIP1) in HEK-293 T cells. **(E)** Bands in **(A–D)** calculated by OD analysis. Data are shown as mean ± SD. **(F–I)** Representative WB bands displaying the co-IP of RAC1 and the MLK3-MKK7-JNK signaling module in the renal cortex. **(J)** The intensity of proteins **(F–I)** is represented as mean ± SD. ^a^*p* < 0.05 vs. the NG group **(E)** and the Con group **(J)**; ^b^*p* < 0.05 vs. the HG group **(E)** and the DM group **(J)**; ^c^*p* < 0.05 vs. the HG + NSC **(E)** group and the DM + NSC group **(J)**. *n* = 4 in each group.

We used Co-IP to examine the interaction between RAC1 and the MLK3-MKK7-JNK signaling module and the scaffold protein JIP1. Co-IP of RAC1/MLK3, RAC1/MKK7, RAC1/JNK, and RAC1/JIP1 was increased in the HG group compared with the NG group, and the RAC1 inhibitor NSC23766 decreased the interaction of RAC1 with the MLK3-MKK7-JNK signaling module (Figures 5A–E). Co-IP of RAC1/MLK3, RAC1/MKK7, RAC1/JNK, and RAC1/JIP1 was enhanced in the DM group compared with the Con group, and co-IP of RAC1 with the MLK3-MKK7-JNK signaling module was blocked by the RAC1 inhibitor NSC23766 (Figures 5F–J). These data suggest that the interaction of RAC1 and the MLK3-MKK7-JNK signaling module play an important role in kidney damage resulting from DN.

### RAC1 Directly Binds to MLK3 in the MLK3-MKK7-JNK Signaling Module

**Figure 6:**
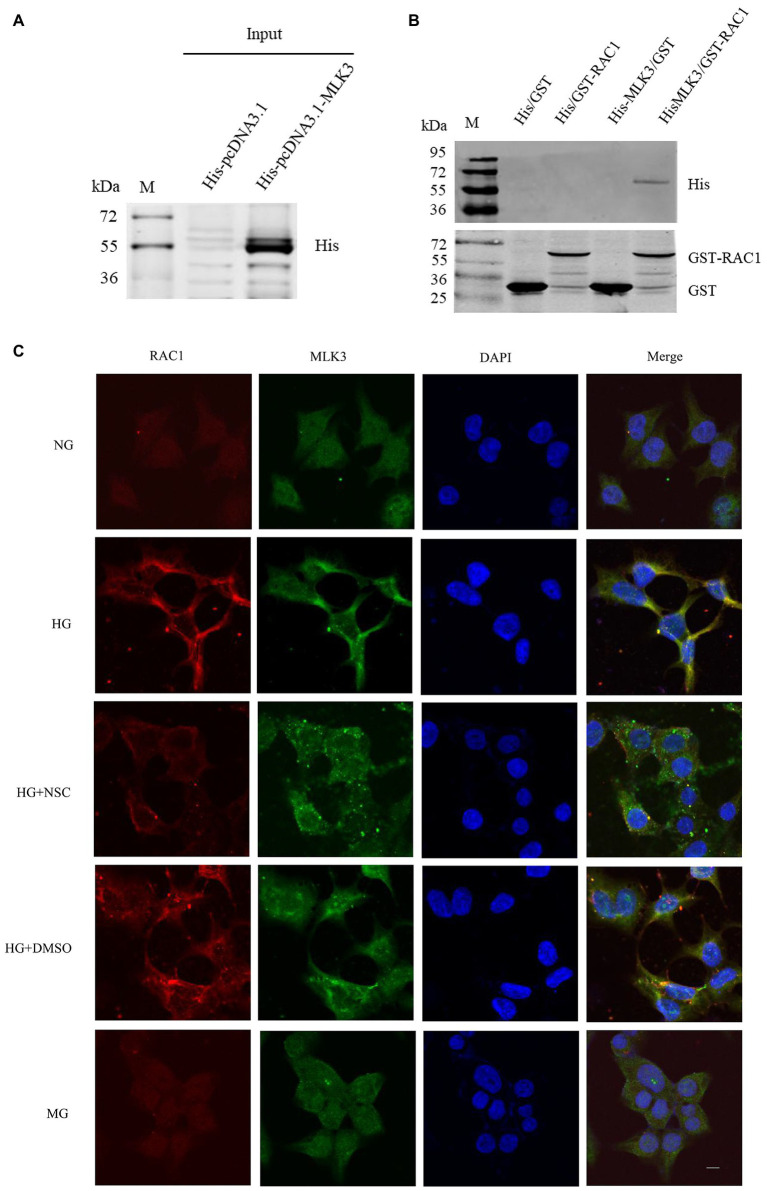
RAC1 interacts directly with MLK3. **(A)** sodium dodecyl-sulfate polyacrylamide gel electrophoresis (SDS-PAGE) and Western blotting (WB) of His-tagged MLK3 fusion protein detected in a pull-down experiment using an anti-His antibody. **(B)** His-tagged MLK3 interacts directly with GST-RAC1 detected by SDS-PAGE and WB with anti-GST and anti-His antibodies. (1) GST as prey protein and His-pcDNA3.1 as bait protein. (2) GST-RAC1 as prey protein and His-pcDNA3.1 as bait protein. (3) GST-PGEX-4 T-1 as prey protein and His-pcDNA3.1-MLK3 as bait protein. (4) GST-PGEX-4 T-1-RAC1 as prey protein and His-FIGURE 6pcDNA3.1-MLK3 as bait protein. **(C)** Colocalization of RAC1 and MLK3. RAC1 is marked in red, MLK3 is marked in green, the cellular nucleus is labeled in blue by DAPI, and the colocalization of RAC1 and MLK3 is presented in yellow. The scale bar is 10 μm (magnification × 400).

We employed GST pull-downs to identify whether RAC1 directly combines with MLK3 in the MLK3-MKK7-JNK signaling module. We built His-pcDNA3.1-MLK3 and GST-PGEX-4T-1-RAC1 fusion protein and control plasmids. GST-RAC1 binds directly to His-MLK3 (Figures 6A,B). These results suggest that RAC1 activates the MLK3-MKK7-JNK signaling module and promotes its assembly through directly combining with MLK3.

### RAC1 Co-locates With MLK3 in the Cytoplasm

To further verify the interaction of RAC1 and MLK3, we investigated the colocalization of RAC1 and MLK3. RAC1 and MLK3 colocalization increased in the HG group compared with the NG group. RAC1 and MLK3 colocalization decreased in the HG + NSC group compared with the HG group (Figure 6C). Consistently, we found that inhibition of RAC1 reduces its interaction with MLK3. These results suggest that high glucose levels promote the colocalization of RAC1 and MLK3 in the cytoplasm, and the RAC1 inhibitor NSC23766 reduces the interaction between RAC1 and MLK3.

### Y40 Is a Critical Site for RAC1 Binding to MLK3

**Figure 7:**
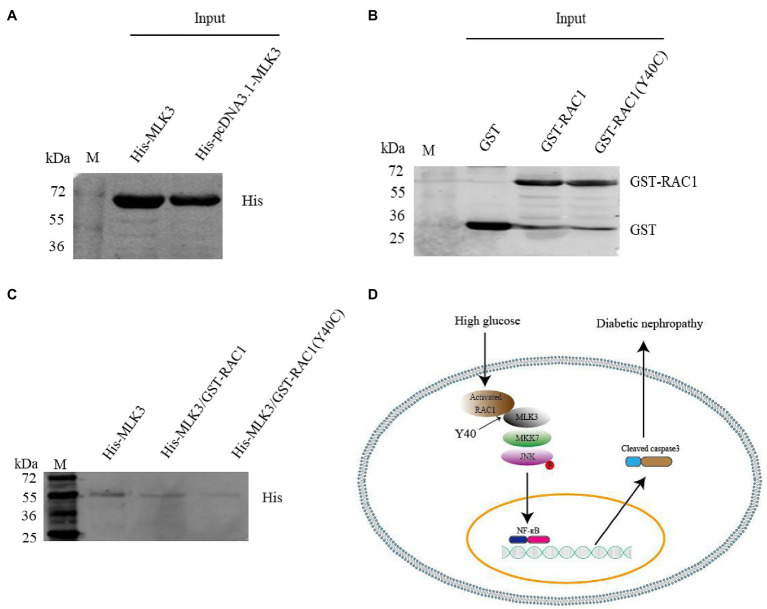
The Y40C mutant inhibits the interaction between RAC1 and MLK3. **(A)** Pull-down experiment showing the detection of His-tagged MLK3 proteins with an anti-His antibody. **(B)** Detection of GST, GST-labeled WT RAC1, and GST-labeled Y40C mutant RAC1 proteins using an anti-GST antibody. **(C)** Interaction of WT RAC1 and Y40C mutant RAC1 with MLK3 tested with an anti-His antibody. (1) Only His-pcDNA3.1-MLK3 as control. (2) GST- PGEX-4 T-1-RAC1 as prey protein and His-pcDNA3.1-MLK3 as bait protein. (3) GST- PGEX-4 T-1-RAC1 (Y40C) as prey protein and His-pcDNA3.1-MLK3 as bait protein. **(D)** Graphical abstract. RAC1, activated by hyperglycemia, directly binds to MLK3 through RAC1 Y40, facilitating MLK3-MKK7-JNK signaling module assembly and JNK phosphorylation, then increases levels of NF-κB and cleaved caspase-3, eventually leading to DN.

The RAC1-acceptor tyrosine is an important site for the interaction of RAC1 with the JNK/MAPK signaling pathway, and the RAC1 Y40C mutant has lost the ability to interact with some Cdc42Hs- or Rac-interacting Binding (CRIB) motif-containing proteins (Meriane et al., 2000; Jiang et al., 2017). Mutating Y40 to C in RAC1 decreased its ability to bind to MLK3 (Figures 7A–C). These data suggest that Y40 is a key site for the RAC1-MLK3 interaction in DN.

## Discussion

In this study, we gained insight into the molecular and cellular mechanisms of RAC1 activation in the progression of DN. The study demonstrates that RAC1 can directly combine with MLK3 to initiate the MLK3-MKK7-JNK signaling module assembly, promoting JNK phosphorylation, NF-κB nuclear transfer, and caspase-3 activation, resulting in apoptosis *in vitro* and *in vivo*, ultimately causing damage to kidney function. Furthermore, we found that Y40 is a key site for the binding of RAC1 to MLK3, and the RAC1 Y40C mutant allows significantly less interaction between RAC1 and MLK3. These results propose a potential novel therapeutic target for DN treatment through targeting RAC1.

Previous studies have shown that high-glucose stimulation can cause RAC1 activation, lead to apoptosis, and aggravate kidney damage (Chowdhury et al., 2019). Diabetes is associated with TG, LDL, BUN, Scr, TC, and 24 h-UP increases, and HDL decrease in people and animals (Kaur et al., 2016; Zheng et al., 2018). Inhibiting RAC1 activation significantly improves biochemical indexes, reduces podocyte apoptosis, and relieves pathological changes in kidney tissue (Van Linthout et al., 2007; Lin et al., 2008). Our results suggest that RAC1 inhibitor NSC23766 improves cell viability. Moreover, NSC23766 significantly decreases apoptosis induced by high glucose. As an eminent nuclear transcription factor, NF-κB plays a critical role in the regulation of many genes involved in apoptosis and inflammation (Baldwin, 1996). Furthermore, NF-κB is tightly controlled by JNK signaling, and cascaded regulation of apoptosis has been demonstrated (De Smaele et al., 2001; Yu et al., 2013). In this study, inhibition of RAC1 significantly blocked JNK/NF-κB signaling, decreased levels of cleaved caspase-3, and exerted an anti-apoptosis effect.

Ras-related C3 botulinum toxin substrate 1 activation is essential for the activation of JNKs (Minden et al., 1995; Matoba et al., 2017). Phosphorylated JNK regulates apoptosis and inflammation (Kyriakis and Avruch, 2012; Nardelli et al., 2018). In consequence, JNK phosphorylation is regulated by the MLK3-MKK7-JNK signaling module, which is indispensable in mechanisms of diabetic kidney injury (Humphrey et al., 2014). As a scaffold protein in the JNK signaling pathway, JIP1 is necessary to maintain or assemble functional JNK activation (Morrison and Davis, 2003). As an upstream signal molecule of JNK, RAC1 promotes the phosphorylation of JNK (Coso et al., 1995). However, whether and how RAC1 induces JNK activation in DN through regulating the MLK3-MKK7-JNK signaling module remains elusive. In this study, the assembly of the MLK3-MKK7-JNK signaling module was increased in DN and significantly decreased with RAC1 antagonist treatment. Moreover, RAC1 could coprecipitate with MLK3, MKK7, JNK, and JIP1 simultaneously. Therefore, RAC1 may upregulate JNK by combining it with the MLK3-MKK7-JNK signaling module in DN. Meanwhile, inhibiting the interaction of RAC1 and the MLK3-MKK7-JNK signaling module could significantly improve cell damage and DN induced by high glucose.

MLK3 contains a CRIB motif, which is capable of binding to RAC1 and disrupting SH3-mediated auto-inhibition, leading to zipper-regulated dimerization and trans-autophosphorylation (Misek et al., 2017). Y40 is an important site through which RAC1 binds to proteins containing a CRIB motif, and the RAC1 Y40C mutant has lost its ability to interact with some CRIB motif-containing proteins (Keller et al., 2005). Our results were in good agreement with other studies, which point to a direct combination of RAC1 and MLK3 *in vitro* (Lambert et al., 2001). Similarly, the results demonstrated that the colocalization of RAC1 and MLK3 increased when induced with high glucose. In addition, Y40, which is located within a bioinformatically predicted RAC1 site, is the target for direct MLK3 binding. The Y40C mutant blocked the interaction of RAC1 and MLK3. Further studies are required to clarify the effect of this RAC1 Y40C mutant on DN in animal models to deeply elucidate the molecular mechanisms involved in activated RAC1 accelerating DN. However, there is still a long way to verify whether this result applies to DN in the clinic. We will used the db/db mice model as the animal model for a type 2 DM and STZ-induced type 1 DM study to illustrate deeply the DN pathogenesis involved in RAC1 further.

## Conclusion

In summary, this study indicates that RAC1 facilitates the occurrence and development of DN through directly combining with MLK3 by virtue of Y40, then promotes MLK3-MKK7-JNK signaling module assembly, and finallt upregulates the JNK signaling pathway (Figure 7D). Inhibition of RAC1 protects against cellular and renal damage induced by high glucose by decreasing MLK3-MKK7-JNK signaling module activation and JNK phosphorylation. This study proves a novel RAC1-related cellular and molecule mechanism of DN, suggesting that prevention and treatment of DN may potentially benefit from new RAC1-targeted treatment strategies.

## Data Availability Statement

The raw data supporting the conclusions of this article will be made available by the authors, without undue reservation.

## Ethics Statement

The animal study was reviewed and approved by Provision and General Recommendation of the Chinese Laboratory Association, and fully complied with the Institutional Animal Care and Use Committee of the Xuzhou Medical College.

## Author Contributions

XZ and YS designed this study. CY, JD, GF, and ZZ conducted the experiments. XZ, CY, JD, and YZ analyzed the results. XZ, CY, JD, ZZ, YZ, and TG wrote this study and reviewed the study. XZ and CY was the guarantor of this study. XZ and YS had full access to all the data in the study and take full responsibility for the data and the accuracy of the data analysis. All authors contributed to the article and approved the submitted version.

### Conflict of Interest

The authors declare that the research was conducted in the absence of any commercial or financial relationships that could be construed as a potential conflict of interest.
